# Ozone Aggravated the Toxicity of Fine Particulate Matter by Impairing Membrane Stability and Facilitating Particle Internalization

**DOI:** 10.3390/toxics13060446

**Published:** 2025-05-28

**Authors:** Jing He, Tong Wang, Han Li, Yemian Zhou, Yun Liu, An Xu

**Affiliations:** 1 Institutes of Physical Science and Information Technology, Anhui University, Hefei 230601, China; 2 Anhui Province Key Laboratory of Environmental Toxicology and Pollution Control Technology, High Magnetic Field Laboratory, HFIPS, Chinese Academy of Sciences, Hefei 230031, China; 3 School of Public Health, Anhui University of Science and Technology, Hefei 231131, China; 4 Science Island Branch, Graduate School of USTC, University of Science and Technology of China, Hefei 230026, China

**Keywords:** fine particulate matter, ozone, joint toxicity, cell membrane damage, detoxification

## Abstract

The combined pollution of fine particulate matter (PM_2.5_) and ozone (O_3_) is increasing synergistically on a global scale, posing a serious threat to human health. However, the joint toxicity and the underlying mechanisms associated with co-exposure to PM_2.5_ and O_3_ remain poorly understood. Through complementary in vivo animal models and in vitro cellular assays, the results demonstrate that although there was no synergistic cytotoxicity effect between PM_2.5_ and O_3_, the presence of O_3_ significantly enhanced the genotoxicity of PM_2.5_ by inducing severe DNA double-strand breaks. Furthermore, O_3_ exposure significantly exacerbated the bioaccumulation of PM_2.5_ by disturbing the cellular membrane integrity, thus leading to synergistic toxicity in bronchial cells and mouse lungs. Astaxanthin (AST) effectively antagonized the adverse effects of PM_2.5_ and O_3_ co-exposure by maintaining cell membrane integrity. These findings enhance our understanding of the pathophysiological mechanisms induced by co-exposure to PM_2.5_ and O_3_, and provide a promising therapeutic strategy for treating respiratory diseases caused by unavoidable exposure to these pollutants.

## 1. Introduction

Air pollution has emerged as one of the most significant global public health challenges in the 21st century [1]. Although many countries have been striving to control the emissions of fine particulate matter (PM_2.5_), the concentration of ozone (O_3_) has gradually increased in recent years, raising the global co-exposure risks of PM_2.5_ and O_3_ [2]. For example, in several Chinese regions burdened by severe PM_2.5_ contamination, atmospheric monitoring data reveal a concomitant increase in O₃ concentration levels [3,4]. From 2018 to 2020, the average annual ozone concentrations in some regions far exceeded China’s Grade 1 standard for O_3_ (100 μg/m^3^) [5]. As a result, air pollution has gradually shifted from a traditional pattern dominated by a single pollutant to a composite pollution pattern [6].

The lung is the main target organ for PM_2.5_ and O_3_ exposure [7,8]. The combination of PM_2.5_ and O_3_ pollution has a synergistic effect on both respiratory disease [9] and the respiratory-related mortality rate [10,11]. Similarly, clinical trials and mouse model studies have demonstrated the synergistic effect in causing pulmonary inflammatory damage [12,13,14], oxidative stress [15], and metabolic disorders [16]. However, although the combined toxicity of PM_2.5_ and O_3_ has been investigated, a systematic understanding of the molecular mechanisms underlying their synergistic toxicity remains unestablished and needs to be deeply explored.

The biological membrane barrier structures within the lung tissue, such as the alveolar epithelial cell layer [17,18], the capillary endothelial cell layer [19], and the basement membrane [20], collectively constitute a critical defense mechanism against the infiltration of exogenous particulate matter. O_3_, as a strong oxidant, can damage the integrity of cell membranes by inducing lipid peroxidation reactions [21], leading to impaired cell membrane function [22]. The disruption of cell membranes facilitates the accumulation process of particulate matter in tissues, and it may further intensify its toxic effects. Therefore, from the perspective of biological barrier integrity, an in-depth interpretation of the mechanism underlying the combined toxicity of PM_2.5_ and O_3_ is of great significance for revealing the biological basis of their synergistic effects.

Maintaining the stability of the plasma membrane could be an effective target for antagonizing the toxic effects of PM_2.5_ and O_3_. Astaxanthin (AST) contains a special structure of 13 unsaturated conjugated double bonds and polar hydroxyl groups at both ends [23]. The unique chemical structure enables AST to be precisely embedded in the phospholipid bilayer, with an excellent effect of enhancing membrane stability [24,25]. Previous research has proven that AST can effectively mitigate cell damage and lung injury induced by PM_2.5_ [26,27], ionizing radiation [28], and heavy metals [29]. Therefore, AST demonstrates great potential in antagonizing the combined toxicity of PM_2.5_ and O_3_.

In this study, we first analyzed the joint toxicity of PM_2.5_ and O₃ and its related effects on Beas-2B cells and mouse lung tissues. The results show that, compared with single exposures to PM_2.5_ or O₃, O₃ pretreatment significantly enhanced the genotoxicity of PM_2.5_ by damaging the cellular membrane and increasing the intracellular deposition of PM_2.5_. Furthermore, by strengthening the cell membrane stability, we demonstrated that AST effectively mitigated the cellular and tissue damage caused by PM_2.5_ and O₃. These findings provide a new research perspective for the in-depth exploration of the combined toxicity mechanisms of air pollutants and their intervention strategies.

## 2. Materials and Methods

### 2.1. Materials

SRM 2975 was selected as the representative PM_2.5_ (NIST, Gaithersburg, MD, USA). O_3_ gas was produced by an O_3_ generator (TONGLINOZONE, Beijing, China) connected to an air generator. The ozone detector (2B Technologies, Boulder, CO, USA) was used to adjust the flow rate to stabilize the concentration in the ozone incubator at specific levels. AST was commercially sourced from Sigma Aldrich (Sigma Aldrich, St. Louis, MO, USA). The AST inclusion complex was prepared using the method in the published work [30].

### 2.2. Size Distribution and Colloidal Stability of PM_2.5_ Particles

PM_2.5_ was diluted to 25, 50, and 100 μg/mL with ultrapure water and Dulbecco’s modified Eagle medium (DMEM) complete medium. The suspension was sonicated and rapidly added to a cuvette for particle size and zeta potential measurements using the Malvern Dynamic Light Scattering instrument (Malvern Panalytical, Malvern, Worcestershire, UK).

### 2.3. Environmental Persistent Free Radicals (EPFRs) Detection

PM_2.5_ was suspended at 50 μg/mL in phosphate buffer saline (PBS) with or without 1 ppm O_3_ pre-treatment for 1 h. EPFRs were characterized using an electron spin resonance spectrometer (Bruker, Rheinstetten, Germany).

### 2.4. Cell Treatment and Exposure

The BEAS-2B cell line was purchased from the Conservation Genetics Kunming Cell Bank of the Chinese Academy of Sciences (Kunming, China). BEAS-2B cells were cultivated at 37 °C and 5% CO_2_ in DMEM (Hyclone, South Logan, UT, USA) supplemented with 10% fetal bovine serum (ExCell Bio, Shanghai, China).

In the experimental design of in vitro and in vivo combined toxicity studies, we pre-designed the exposure sequence based on the spatial and temporal distribution characteristics of atmospheric pollutants—O_3_ pre-exposure, followed by PM_2.5_ exposure. This design is based on the following scientific rationale. Specifically, the sources of PM_2.5_ include direct emissions and secondary production (e.g., photochemical oxidation), whose concentrations increase significantly under stable meteorological conditions (e.g., nighttime to early morning when the boundary layer is low) [31,32,33]. As a typical photochemical secondary pollutant, O_3_ concentrations peak during periods of intense sunlight, and its generation is dependent on the photochemical reaction of precursors [34].

The study comprised seven experimental groups, as follows: control (untreated); solvent control (vehicle only); AST (10 μM); PM_2.5_ (25, 50, 100 μg/mL, 24 h); O_3_ (0.2, 0.4, 0.6, 0.8, 1 ppm, 1 h in PBS followed by 24 h medium culture); PM_2.5_ + O_3_; PM_2.5_ + O_3_ + AST. For O_3_ exposure, cells were incubated in PBS to minimize medium ion interference, with subsequent replacement by fresh medium for continued culture. Co-exposure groups received sequential treatments—initial O_3_ preconditioning (1 ppm, 1 h), followed by PM_2.5_ (50 μg/mL) or AST (10 μM) administration for 24 h. The concentrations of PM_2.5_ (25, 50, and 100 μg/mL) and O_3_ (0.2, 0.4, 0.6, 0.8, and 1 ppm) used in this experiment were determined based on the existing references [35,36] as well as data from heavily contaminated areas [4], and taking into account the species differences between humans and rodents, whose tolerance to pollutants is four to five times higher than that of humans [37,38].

### 2.5. Cytotoxicity Testing

The CCK8 assay (APExBIO, Houston, TX, USA) was used to test the cell viability of BEAS-2B cells in response to different concentrations of O_3_. Briefly, cells (5 × 10^3^ cells/well) were plated into 96-well plates (NEST Biotechnology, Nanjing, China) and cultured for 24 h. After cell treatment, 10% CCK8 reagent was introduced to each well for a 4 h incubation and assayed at an absorbance wavelength of 450 nm.

The clonal survival assay was employed to detect the cytotoxicity of PM_2.5_ and co-exposure with O_3_ and AST. BEAS-2B cells were seeded in 60 mm transparent culture dishes (Jet Biofil, Guangzhou, China). After treatments, cells were passaged to new 60 mm culture dishes (600 cells per dish) and incubated for 7–10 days. After a clear cell population appeared, we discarded the old solution, washed it with PBS, and fixed it with a fixative reagent (methanol: glacial acetic acid = 9:1) for 20 min. Next, cells were dyed with crystal violet for 4 h and washed gently under running water. Finally, the clone points at the bottom were counted and statistically analyzed using optical microscopy (Olympus CK2, Tokyo, Japan).

### 2.6. Western Blotting

As mentioned in the previous study [39], proteins were extracted with RIPA buffer (Biosharp, Heifei, China) and quantified using a commercial kit (Thermo Fisher Scientific, Waltham, MA, USA). The proteins were electrophoresed on an SDS-PAGE gel (YaMei, Shanghai, China) and then transferred to a PVDF membrane (Roche, Basel, Switzerland). The membranes were then sealed with a protein-free rapid sealing buffer (YaMei, Shanghai, China). After sealing, the PVDF membrane was immersed in primary antibodies γ-H2AX (Millipore, Billerica, MA, USA) and β-actin (ZSGB-Bio, Beijing, China), and then incubated with secondary antibody (Promega Corporation, Madison, WI, USA). Protein bands were detected and quantified through chemiluminescent signal acquisition with the Tanon-5200 imaging system (Tanon, Shanghai, China), and the results were analyzed using ImageJ (Fiji/ImageJ 2.0, Dresden, Germany).

### 2.7. Membrane Potential Assay

BEAS-2B cells were cultured in opaque 96-well microplates (Labselect, Beijing, China) at a density of 5 × 10^3^ cells per well. When the cells reached 80% confluency (logarithmic growth phase), they were treated following predefined group assignments. Subsequently, each group was incubated with 10 μM DiBAC4 (3) under controlled conditions (37 °C, 5% CO_2_) for 30 min. Fluorescence signals were quantified at Ex/Em 493/516 nm using a SpectraMax i3 multi-functional detection platform (Molecular Devices, Sunnyvale, CA, USA).

### 2.8. Membrane Rupture Assay

Plasma membrane integrity was assessed by quantifying lactate dehydrogenase (LDH) release into the extracellular medium. After the cell treatment, cells were harvested in sterile centrifuge tubes containing complete culture medium and centrifuged to pellet cellular debris. The LDH release in the cell supernatant was detected using a commercial cytotoxicity assay kit (NJJCBIO, Nanjing, China). Absorbance values were recorded at 440 nm with a SpectraMax i3 multi-functional detection platform (Molecular Devices, Sunnyvale, CA, USA).

### 2.9. Ca^2+^ Flux Assay

Intracellular Ca^2+^ flux was detected by Fluo-3/AM fluorescent probe (Beijing Solarbio Science & Technology Co., Ltd., Beijing, China). The procedure for the Ca^2+^ flux assay was revised as follows: Cells were inoculated into black 96-well plates and processed after wall attachment. They were then incubated with 5 μM Fluo-3/AM for 30 min in the absence of light. Then, the cells were washed three times by HBSS and supplemented with 100 μL of HBSS to continue the transformation away from light for an additional 30 min, and the fluorescence intensity was detected by a SpectraMax i3 multi-functional detection platform (Molecular Devices, Sunnyvale, CA, USA) using an enzyme marker (Ex/Em = 488/530 nm).

### 2.10. Observation of the Morphology of PM_2.5_ and the Localization of PM_2.5_ in Cells by TEM

As mentioned in the previous study [40], PM_2.5_ was dispersed in ultrapure water and treated with ultrasonic waves to make it evenly dispersed. Then, 10 μL of the suspension was taken and dripped uniformly onto a copper mesh and left to adsorb for 2 min, before being allowed to dry. After drying, it was loaded into a feed bin and observed and photographed with a TEM (JEM2100 Plus, JEOL Ltd, Tokyo, Japan).

TEM observation in cells was performed as described [41]. After cell exposure, cells were rinsed with glutaraldehyde (TED PELLA, Inc., Redding, CA, USA) and collected. The collected samples were fixed, embedded, dehydrated, infiltrated, and then sectioned on a microtome (Leica UC-7, Wetzlar, Germany). High-resolution TEM imaging was performed on a JEM-1400 platform (JEOL Ltd., Tokyo, Japan) equipped with a Morada G3 digital acquisition unit, which was used to observe the cellular ultrastructural characteristics.

### 2.11. Animal Experimentation

In vivo experiments were conducted using male-specific pathogen-free C57BL/6J mice (Zhejiang Vital River, Zhejiang, China). The barrier maintains an ambient temperature of 22 ± 1 °C, a relative humidity of 50 ± 10%, and 12 h of alternating day and night light; the single-cage rearing density was no more than 5, guaranteeing the frequency of ≥50 cage air changes per hour; the noise level was maintained at less than 60 dB, the concentration of ammonia was strictly controlled within the threshold value of 14 ppm, and all the indexes complied with the national standards (GB 14925-2023) for environmental facilities with experimental animals [42]. Microbial control is achieved through SPF-level pathogen monitoring, contact sterilization and personnel decontamination. Laboratory animals have free access to sterilized feed and drinking water, and cage bedding is changed regularly to ensure hygiene. Before experimental procedures, mice were allowed a 7-day acclimation period to minimize environmental stress.

The mice were randomly assigned to seven groups, as follows (*n* = 5): control, solvent control, AST (2 mg/kg, tracheal instillation for 2 weeks, once a day), PM_2.5_ (5 mg/kg, tracheal instillation for 2 weeks, once a day), O_3_ (1 ppm for 3 h once a day for 2 weeks), PM_2.5_ + O_3_ (1 ppm for 3 h, and then intratracheally administered with 5 mg/kg PM_2.5_ in the mice once a day for 2 weeks), and PM_2.5_ + O_3_ + AST (mice were pre-exposed with 1 ppm O_3_ for 3 h, and we then simultaneously instilled 5 mg/kg of PM_2.5_ and 2 mg/kg of AST through the trachea once a day for two weeks). All the animal studies complied with the Animal Research: Reporting of In Vivo Experiments (ARRIVE) guidelines, the National Research Council’s Guide for the Care and Use of Laboratory Animals, and the Chinese Guideline for Ethical Review of Animal Welfare in Laboratory Animals. Ethical approval for this study was obtained from the Ethics Committee of the Institute of Health and Medicine, Hefei Comprehensive National Science Center (Approval Number: IHM-AP-2023-003, Date: 17 June 2023).

### 2.12. Histopathology Detection

The lung sections were stained following the previously outlined method [43]. HE staining was revised as follows: Mouse lung tissues were immersed in 4% paraformaldehyde fixative for at least 1 day at the end of the experiment. Subsequently, the tissues were embedded following a gradient ethanol multi-step dehydration process, xylene treatment, and paraffin dip waxing. Next, the tissues were cut into approximately 5 μm-thick sections using a sectioning machine and stained with hematoxylin and eosin (H&E). The staining process included the deparaffinization of the sections, gradient ethanol treatment, hematoxylin staining, differentiation in 1% hydrochloric acid alcohol solution, bluing in dilute lithium carbonate aqueous solution, eosin dye staining, and finally treating the sections with anhydrous ethanol and xylene. The sections were sealed with neutral resin, dried naturally, subjected to microscopic observation and photographed for recording.

### 2.13. Determination of Inflammatory Cytokines

After exposure, mice were euthanized, and their blood was collected and centrifuged to obtain serum (2000× *g*, 4 °C, 20 min). The concentrations of inflammatory factors IL-1β and TNF-α in the serum were measured using commercial kits (Cusabio, Wuhan, China). The assay was performed in strict accordance with the steps in the instructions [35].

### 2.14. Statistical Analysis

Statistical processing was conducted through GraphPad Prism 10 (Version 10.0.0, GraphPad Software Inc., San Diego, CA, USA), implementing parametric analysis via one-way ANOVA and two-way ANOVA with Tukey’s post hoc analysis. All the data are represented as mean ± standard deviation (SD) of at least three independent replicates. A *p*-value < 0.05 was regarded as indicating statistical significance.

## 3. Results and Discussion

### 3.1. Characterization of PM_2.5_

**Figure 1:**
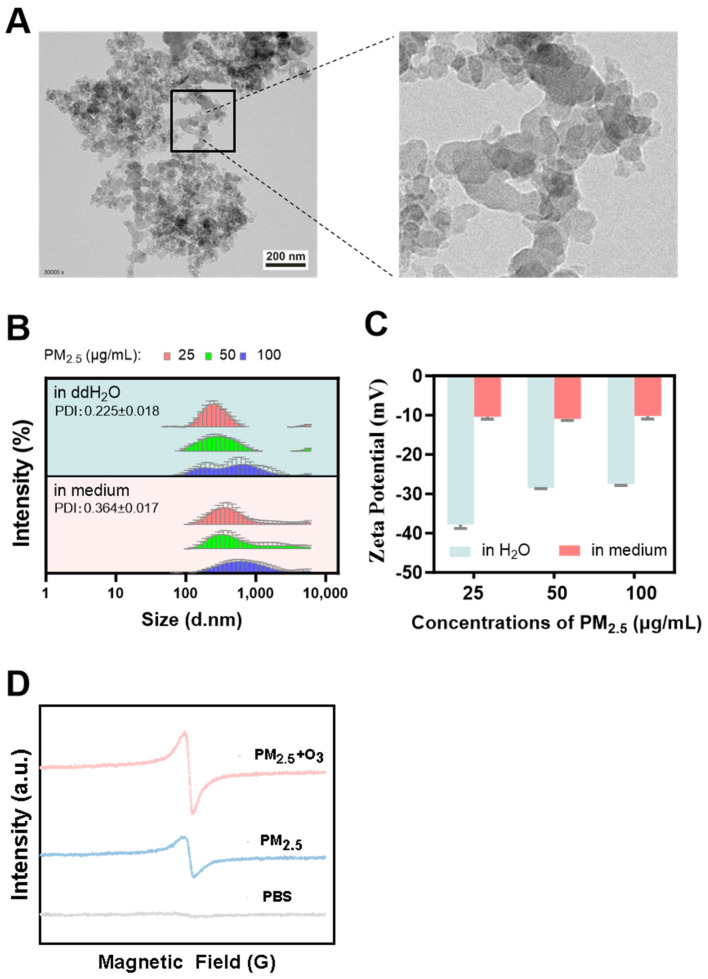
Characterization of PM_2.5_ at varying concentrations and in different media. (**A**) TEM images of PM_2.5_. Scale bar = 200 nm. (**B**) The average hydrated particle sizes and (**C**) zeta potentials for 25, 50, and 100 μg/mL PM_2.5_ in different media. (**D**) EPR spectra of EPFRs in PM_2.5_ with or without O_3_ pre-treatment. Blue line: EPR spectra of 50 μg/mL PM_2.5_. Red line: EPR spectra of 50 μg/mL PM_2.5_ after 1 ppm O_3_ pre-treatment (1 h). Grey line: EPR spectra of PBS used as a control solution.

Numerous studies have shown that PM’s physical and chemical properties directly affect its toxicological effects [44,45]. As shown in Figure 1A, TEM images revealed that the typical morphology of PM_2.5_ was soot particles with a size of less than 50 nm. Although larger agglomerates of several microns could be formed, most particles were less than 1 μm. Dynamic Light Scattering (DLS) was used to measure the hydrated particle size and zeta potential of PM_2.5_ dispersed in ultrapure water and cell culture medium [46]. As shown in Figure 1B, the average hydrated particle sizes of 25, 50, and 100 μg/mL PM_2.5_ in water were 258.1 ± 3.496 nm, 276.8 ± 7.225 nm, and 356.9 ± 8.143 nm, respectively. The average hydrated particle sizes of these concentrations of PM_2.5_ in culture medium were 352.2 ± 5.294 nm, 400.9 ± 10.43 nm, and 525.3 ±6.198 nm, respectively. The relatively larger particle size of PM_2.5_ in the culture medium might be attributed to the fact that, as a kind of nanoparticle, certain protein molecules in the medium could promote the aggregation of the particles [47]. Zeta (ζ) potential refers to the strength of repulsion or adsorption between particles, and is an essential indicator used for characterizing the stability of colloidal dispersions. As depicted in Figure 1C, the surface charges of PM_2.5_ were all negative, either in water or in cell culture medium, while the absolute values were higher in water than in culture medium, which also indicates the result that PM_2.5_ was more prone to agglomerate in the culture medium [48].

Compared to short-lived free radicals [49], EPFRs can stably exist within particles for extended periods, ranging from hours to days, and even months [50,51], thus posing more severe adverse effects on living organisms. Our results show that a significant EPFR signal was detected in PM_2.5_ with or without O_3_ co-treatment, and O_3_ elevated the mean concentration of EPFR in PM_2.5_. The signal intensity of the PM_2.5_ and O_3_ co-exposure group was approximately 1.76-times higher than that of the PM_2.5_ exposure group (Figure 1D). As a complex pollutant in the atmosphere, PM_2.5_ adsorbs many organic and inorganic substances on its surface, providing abundant reaction precursors for generating EPFRs [52]. O_3_ was reported to react with organic pollutants (e.g., polycyclic aromatic compounds) to promote the formation of EPFRs [53]. Our results suggest that the enhanced production of EPFRs on PM_2.5_ in the presence of O_3_ likely leads to severe oxidative damage to living organisms.

### 3.2. Cytotoxicity and Genotoxicity Induced by PM_2.5_ and O_3_

**Figure 2:**
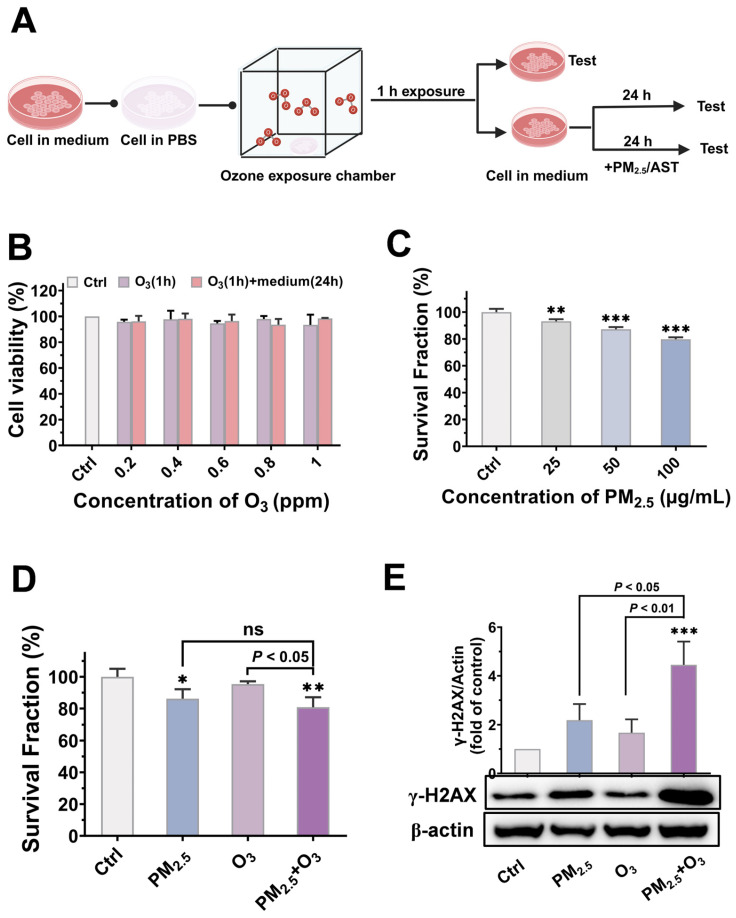
Cytotoxicity and genotoxicity of PM_2.5_ and O_3_. (**A**) Experimental flowchart. (**B**) The dose-dependent changes in cellular viability induced by O_3_. Purple column: BEAS-2B cells were exposed to 0.2, 0.4, 0.6, 0.8, and 1 ppm O_3_ for 1 h. Pink column: cells were maintained in culture conditions for another 24 h after O_3_ exposure (1 h). (**C**) The survival fraction of BEAS-2B cells exposed to different concentrations of PM_2.5_ (25, 50, and 100 μg/mL) for 24 h. The combined effects of O_3_ and PM_2.5_ on (**D**) survival fraction and (**E**) γ-H2AX protein levels of BEAS-2B cells. Cells were treated with PM_2.5_ (24 h) or O_3_ (1 h in O_3_ + 24 h in culture condition) or combined-treated with 1 ppm O_3_ for 1 h, and this was immediately followed by 50 μg/mL PM_2.5_ exposure for another 24 h. *, *p* < 0.05; **, *p* < 0.01; ***, *p* < 0.001, compared to control group; ns, no significance.

Earlier investigations predominantly focused on the negative consequences of single environmental contaminants. At the same time, limited research has investigated the joint toxicity of PM_2.5_ and O_3_ to bronchial cells. Therefore, the cytotoxicity of PM_2.5_ and O_3_ at graded concentrations was first tested in BEAS-2B cells to identify an appropriate concentration for the subsequent experiments. Considering that O_3_ exposure in daily life is typically short-term and has been shown to induce not only acute hazards but also progeny damage [54,55], the effects caused by O_3_ were detected either immediately following a 1 h treatment, or after a 1 h treatment followed by a 24 h recovery period in the cell culture medium; the experimental procedure is depicted in Figure 2A.

No significant differences were observed between non-exposed cells and cells exposed to O_3_ at varying concentrations or during different recovery phases (Figure 2B). Consequently, a concentration of 1 ppm was selected for O_3_ in the following experiments. To avoid the influence of material color on absorbance measurements, the clone survival method was employed to detect the cytotoxicity of PM_2.5_. The cell proliferation ability in cells treated with PM_2.5_ decreased gradually with the increase in exposure concentrations, indicating a dose-dependent cytotoxicity of PM_2.5_ (Figure 2C). A concentration of 50 μg/mL PM_2.5_ was used for the following study. Compared to PM_2.5_ treatment alone, a slight toxicity elevation could be found in cells co-treated with O_3_ and PM_2.5_ (the survival fraction decreased from 86.23 ± 5.95% to 80.86 ± 6.26%, *p* > 0.05) (Figure 2D), indicating that compared to PM_2.5_ treatment alone, there was no significant joint cytotoxicity when cells were co-exposed to O_3_ and PM_2.5_.

DNA double-strand breaks (DSBs) were chosen as a typical marker of genetic damage [56]. Although there was no obvious cytotoxicity when cells were treated with 1 h of exposure to O_3_ followed by 24 h of recovery, slight DNA damage could be observed in O_3_-treated cells, which could not be completely repaired. The Western blotting results also indicate that O_3_ preincubation enhanced the genotoxicity of PM_2.5_, as the protein expression of γ-H2AX was increased by approximately 100% (Figure 2E). PM_2.5_ has been recognized as a definitive human carcinogen [57], while O_3_ has been reported to attack DNA molecules [58]. The synergistic genotoxicity of PM_2.5_ and O_3_ has aroused significant health concerns in the context of current atmospheric composite pollution, highlighting the need for further elucidation of the underlying mechanisms involved.

### 3.3. O_3_ Pre-Treatment Enhanced the Toxicity of PM_2.5_ via Destroying the Plasma Membrane Integrity and Facilitating the Bioaccumulation of PM_2.5_

**Figure 3:**
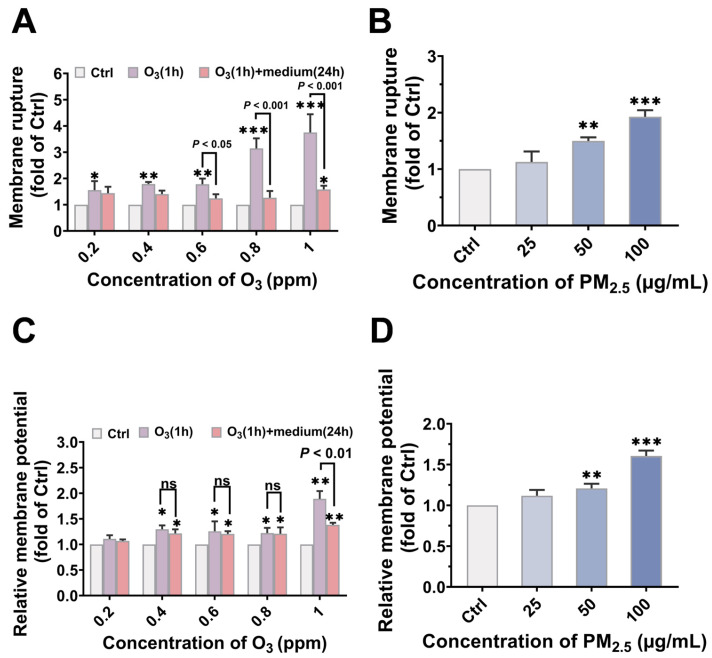
Membrane damage caused by PM_2.5_ and O_3_, respectively. The dose-dependent changes in (**A**) LDH release and (**C**) membrane potential induced by O_3_. Purple column: BEAS-2B cells were exposed to 0.2, 0.4, 0.6, 0.8, and 1 ppm O_3_ for 1 h. Pink column: cells were maintained in culture conditions for another 24 h after O_3_ exposure (1 h). The (**B**) LDH release and (**D**) membrane potential of BEAS-2B cells to 0, 25, 50, and 100 μg/mL of PM_2.5_ for 24 h. *, *p* < 0.05; **, *p* < 0.01; ***, *p* < 0.001, compared to control group; ns, no significance.

Plasma membrane integrity, the first and primary protective barrier against contamination invasion, is critical for maintaining cellular stability [59]. PM_2.5_ and O_3_ have been reported to disrupt membrane stability [60,61]. Therefore, we next interpreted the mechanism involved in the joint toxicity of PM_2.5_ and O_3_ from the point of view of plasma membrane integrity. Membrane rupture and relative membrane potential were used to evaluate the cell membrane integrity. As shown in Figure 3, significant LDH release and membrane potential elevation were observed in both O_3-_ and PM_2.5_-treated cells, indicating a dose-dependent effect on cell membrane damage. However, the disruption caused by 1 h O_3_ pre-treatment could be restored after 24 h of recovery. Specifically, LDH release increased with increasing O_3_ concentration, from 1.56 ± 0.34 times at 0.2 ppm to 3.75 ± 0.69 times at 1 ppm. In contrast, after 24 h of recovery, the level of LDH release induced by 1 ppm O_3_ decreased from 3.75 ± 0.69 to 1.58 ± 0.14 (*p* < 0.001) (Figure 3A), which was probably due to the self-repair ability of the cell membrane in response to O_3_ exposure through lipid metabolism, protein synthesis and cell membrane fusion [62].

**Figure 4:**
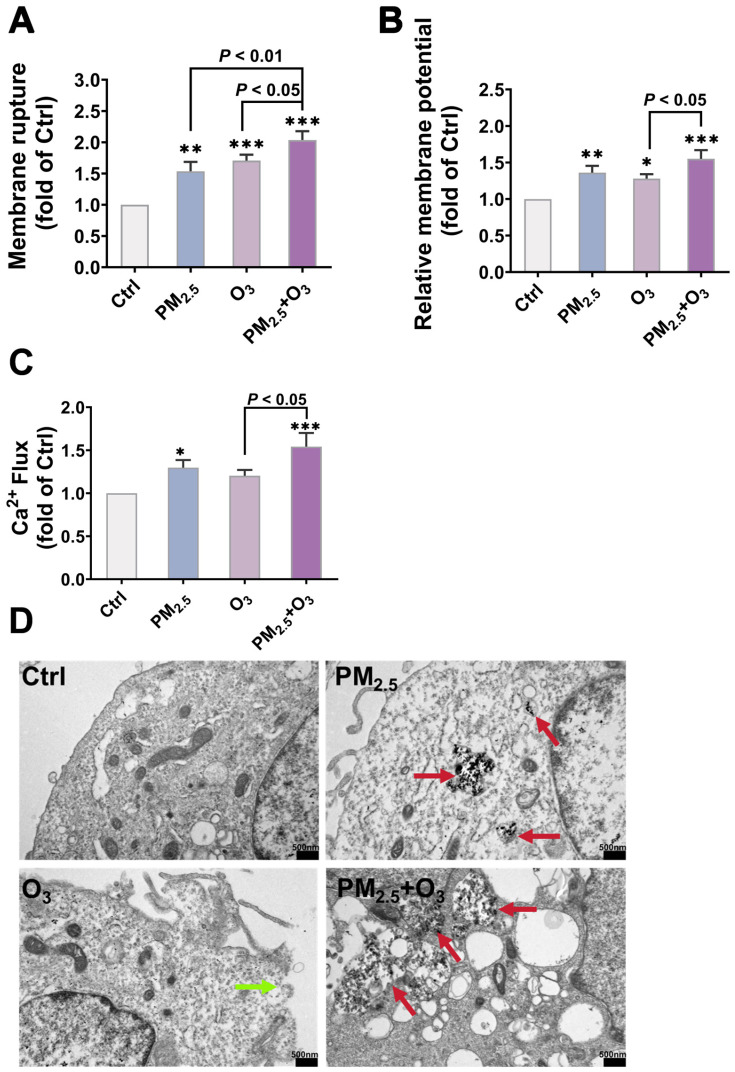
Combined effects of O_3_ and PM_2.5_ on membrane damage and PM_2.5_ bioaccumulation. (**A**) LDH release, (**B**) membrane potential, and (**C**) Ca^2+^ flux. (**D**) Direct observation of cell membrane rupture (green arrows) and PM_2.5_ bioaccumulation (red arrows) in BEAS-2B cells. Scale bar = 500 nm. Cells were pre-treated with O_3_ (1 h in O_3_ + 24 h in culture conditions) or PM_2.5_ (24 h), or combined-treated with 1 ppm O_3_ for 1 h, followed by 50 μg/mL PM_2.5_ treatment for another 24 h. *, *p* < 0.05; **, *p* < 0.01; ***, *p* < 0.001, compared to the control group.

Compared with the groups exposed to PM_2.5_ alone and O_3_ alone, the combined exposure to PM_2.5_ and O_3_ increased the release of LDH in the cell supernatant by 1.33 ± 0.04 times (*p* < 0.01) and 1.19 ± 0.03 times (*p* < 0.05), respectively (Figure 4A). Combined exposure also significantly increased the plasma membrane depolarization compared with the control, PM_2.5_ alone, and O_3_ alone groups (Figure 4B). Ca^2+^ is an important and ubiquitous second messenger that regulates various cellular processes and maintains cellular homeostasis [63,64]. The excessive influx and uptake of Ca^2+^ into the cytoplasm imply cellular stress, and can lead to cellular overload, resulting in cell death. As shown in Figure 4C, co-exposure resulted in a pronounced increase in intracellular Ca^2+^ levels, reaching 1.54 ± 0.09 times that of the control group (*p* < 0.001) and 1.28 ± 0.06 times that of the O_3_-only exposure group (*p* < 0.05).

The damage to the cellular membrane was further confirmed intuitively by TEM observation. As shown in Figure 4D, cells in the control group presented a typical normal structure of BEAS-2B cells with an intact plasma membrane and organelles in good condition. Significant membrane rupture on the cell surface was observed in the O_3_-exposed group (green arrow), indicating a disruptive role of O_3_ on cell membrane integrity. Furthermore, compared to the PM_2.5_-treatment group, a significantly greater number of particle aggregates (red arrows) were deposited in the cytoplasm of cells under combined exposure, suggesting that O_3_ pre-treatment disrupted the integrity of the cellular membrane and promoted the bioaccumulation of PM_2.5_, which probably contributed to the synergistic toxicity of combined exposure to PM_2.5_ and O_3_.

PM_2.5_ harbors complex toxic constituents [65,66], capable of inducing membrane destabilization via multi-modal pathways. Furthermore, EPFRs present in PM_2.5_ can act as electron donors to generate reactive oxygen species (ROS), which attack lipid molecules in the cell membrane, triggering lipid peroxidation and damaging the cell membrane [67]. O_3_ is a strong oxidant, and it can significantly damage the structure and the normal function of cell membranes [68]. Previous studies have proven the synergistic effects of PM_2.5_ and O_3_ co-exposure [69,70]. However, these studies have not yet fully revealed the specific mechanisms of damage to cell membranes caused by combined pollutants. In this study, we initiated an investigation from a novel perspective focused on membrane damage induced by PM_2.5_ and O_3_ co-exposure, achieving a multidimensional understanding of the combined toxic effects of PM_2.5_ and O_3_. In our study, on the one hand, we found that the presence of O_3_ increased the loading of EPFRs in PM_2.5_; this process may enhance the stability of free radicals within PM_2.5_ through heterogeneous reactions with surface organic matter, thereby extending their half-life [71]. On the other hand, the presence of O_3_ led to the breakage of the cell membrane and exacerbated the accumulation of PM_2.5_ in the cell. Therefore, decreased plasma membrane stability was considered to be an important mechanism involved in the combined toxicity of PM_2.5_ and O_3_ in our study. In the subsequent work, we will further focus on the mechanisms by which O_3_ enhances the loading of EPFRs in PM_2.5_, and the mechanisms of EPFRs in inducing cell membrane damage and joint toxicity.

### 3.4. AST Mitigated the Adverse Effects Caused by PM_2.5_ and O_3_ Co-Exposure

**Figure 5:**
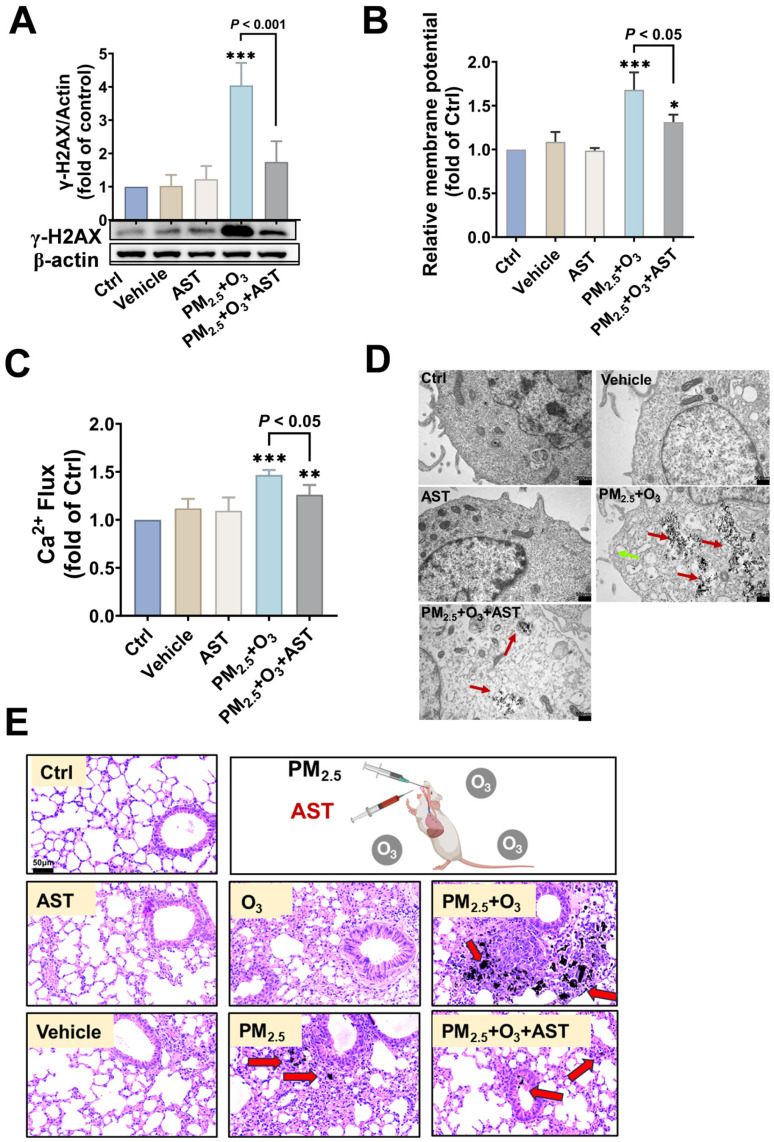
Suppression of PM_2.5-_ and O_3_-induced toxicity by AST in vitro and in vivo. (**A**) γ-H2AX protein levels, (**B**) membrane potential, and (**C**) Ca^2+^ flux in cells treated with AST (10 μM) and 50 μg/mL PM_2.5_ for 24 h after pre-treatment with 1 ppm O_3_ for 1 h. (**D**) Direct observation of the protective role of AST on cell membrane breakage (green arrow) and PM_2.5_ bioaccumulation (red arrows) induced by PM_2.5_ and O_3_ co-exposure. Scale bar = 500 nm. (**E**) H&E of lung tissues collected from PM_2.5_, O_3,_ and AST-treated mice. Scale bar = 50 µm. Red arrows indicate PM_2.5_ particles. *, *p* < 0.05; **, *p* < 0.01; ***, *p* < 0.001, compared to control group.

Previous studies have shown that AST has the function of maintaining membrane stability [72]. We thus hypothesized that AST could inhibit the toxicity of PM_2.5_ and O_3_ co-exposure by preserving membrane integrity. As shown in Figure 5A, the addition of AST significantly inhibited the DNA damage elicited by PM_2.5_ and O_3_ co-exposure, with the protein expression of γ-H2AX decreasing from 4.04 ± 0.68 times to 1.75 ± 0.62 times (*p* < 0.001). Furthermore, co-treatment with AST sharply inhibited the LDH release induced by PM_2.5_ and O_3_ co-treatment from 1.88 ± 0.09 times to 1.17 ± 0.08 times (*p* < 0.05), nearly to the base level of the control group (Supplemental Files, Figure S1). The elevated membrane potential (Figure 5B) and Ca^2+^ influx (Figure 5C) induced by PM_2.5_ and O_3_ co-treatment also declined after AST addition, indicating a protective role of AST in preserving cellular membrane integrity. The alteration of PM_2.5_ bioaccumulation was further verified by TEM detection. Cellular membrane rupture (green arrow) and massive PM_2.5_ aggregates (red arrows) could easily be observed in cells co-treated with PM_2.5_ and O_3_. Meanwhile, the microvilli on the cell membrane surface disappeared, and numerous intracellular vesicles formed within the cell. In contrast, AST co-treatment maintained the integrity of the cell membrane and inhibited the bioaccumulation of PM_2.5_ in the cytosol (Figure 5D). These data suggest that AST antagonized the toxicity of PM_2.5_ and O_3_ by maintaining cellular membrane integrity and reducing the bioaccumulation of PM_2.5_.

In vivo experiments also confirmed the remarkable antagonistic effect of AST. As one of the organs directly interacting with the external environment, the surface of the lung is lined with a complex layer of epithelial tissue that harbors immune cells, forming the first line of pulmonary defense [73]. They effectively prevent further lung damage by identifying and eliminating inhaled foreign substances, pathogens, and pollutants [74,75]. PM_2.5_ could penetrate the body’s multiple defense mechanisms, deposit in the alveoli, and accumulate in lung fluids [76]. Smaller particles can even penetrate the air–blood barrier, enter the circulation, and pose potential health risks to other organs in the body [77]. To further confirm the effect of AST against the negative impacts caused by PM_2.5_ and O_3_, we evaluated the effects in mice. Figure 5E shows the degree of damage in the lung tissues of mice. Histopathological analysis revealed a preserved alveolar architecture and intact interstitial matrices in control and AST-administered cohorts. Notably, the PM_2.5_ and O_3_ co-exposure group demonstrated synergistic pathological exacerbations, exhibiting intensified vascular endothelial swelling, increased alveolar type II epithelial cells, and inflammatory exudate in the alveoli. The existence of O_3_ significantly exacerbated the deposition of PM_2.5_ in the lung, as indicated by the red arrows. However, AST restored the structural damage of the lung and decreased the PM_2.5_ accumulation. Inflammation, as a vital response to external stimuli, has been identified as a primary systemic mechanism underlying the adverse effects caused by air pollutants. TNF-α and IL-1β (pro-inflammatory cytokines) levels in the mice serum exposed to PM_2.5_ and O_3_ were significantly increased compared with the control group (Supplemental Files, Figure S2). Previous findings have demonstrated that combined exposure to urban particulates and O_3_ exacerbated cellular damage and interstitial inflammation in rat lungs, suggesting that the two pollutants may have more potent toxic effects when present together [78]. However, in the AST administration group, the levels of TNF-α (*p* < 0.001) and IL-1β (*p* < 0.05) were decreased considerably. Although previous studies have reported the anti-inflammatory [79] and antioxidant properties of AST [80], few studies have focused on the inhibitory effect of AST on the combined toxicity of atmospheric complex pollutants, especially PM_2.5_ and O_3_. This study has identified that AST also had a good inhibitory effect on the toxicity of PM_2.5_ and O_3_ combined exposure, especially in inhibiting DNA damage, repairing membrane stability, and exerting anti-inflammatory effects.

Although experiments have demonstrated the damage caused by PM_2.5_ and O_3_ to cell membranes and the excellent antagonistic effect of AST on combined exposure, several areas still deserve in-depth exploration in existing research. Currently, most experiments are still limited to cell models and in vivo lung tissue, and systematic studies on the interactions of multiple organs are still relatively scarce. For example, pollutants can enter the blood, brain, placenta, etc., through the blood–air barrier, and further in-depth exploration is still needed.

## 4. Conclusions

In summary, the results of this study indicate that O_3_ exacerbated the toxic effects of PM_2.5_ by disrupting the stability of cell membranes, leading to the excessive deposition and retention of PM_2.5_ within cells and in the lungs. Regarding prevention and detoxification, we emphasize the significant role of AST in counteracting the toxic effects caused by PM_2.5_ and O_3_ co-exposure. Based on these preliminary studies, future research can further explore the impacts of complex atmospheric pollutants on the body under various conditions, providing a scientific basis for risk assessment and developing prevention and detoxification approaches for air pollution.

## Data Availability

All the datasets generated for this study are included in the article.

## Supplementary Materials

The following supporting information can be downloaded at: https://www.mdpi.com/article/10.3390/toxics13060446/s1, Figure S1: LDH release; Figure S2: Levels of (A) IL-1β and (B) TNF-α in mouse serum treated by PM_2.5_, O_3_, and AST.
