# Supplementary material for: Ozone Aggravated the Toxicity of Fine Particulate Matter by Impairing Membrane Stability and Facilitating Particle Internalization
Source: Toxics. 2025 May 28;13(6):446. doi: 10.3390/toxics13060446 (PMC12197588; doi:10.3390/toxics13060446)
Supplement: Supplementary file 1 [file toxics-13-00446-s001.zip › toxics-3628311-supplementary.pdf]

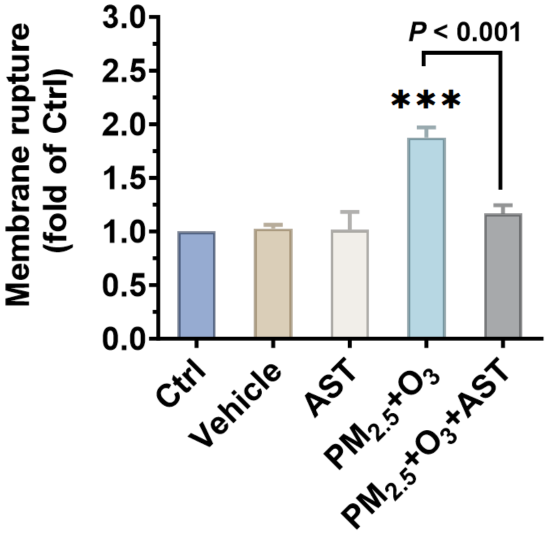

Figure S1. LDH release. Cells treated with AST (10 μM) and 50 μg/mL $PM_{2.5}$ for 24 h after pre-treated with 1 ppm O_3 for 1 h. *: $P < 0.05$, **: $P < 0.01$, ***: $P < 0.001$, compared to the control group.

A

B

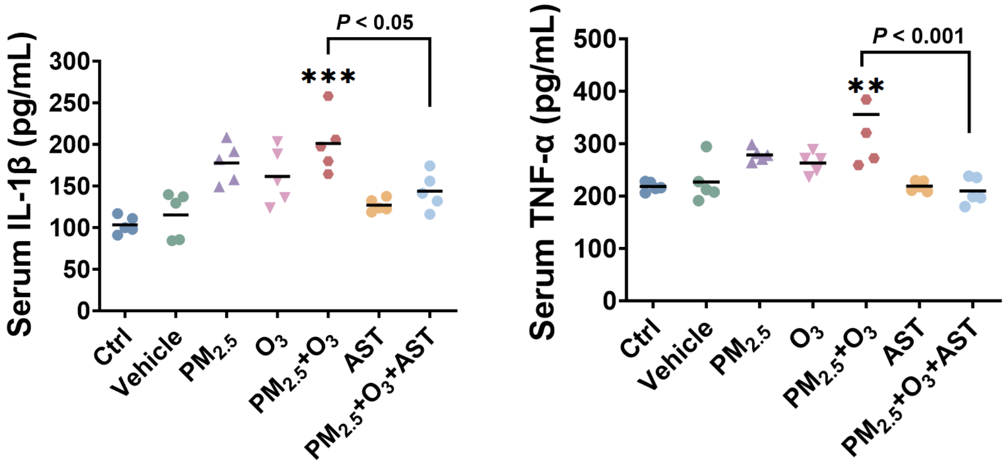

Figure S2. Levels of (A) IL-1β and (B) TNF-*α* in mouse serum treated by $PM_{2.5}$, O_3, and AST. *: $P < 0.05$, **: $P < 0.01$, ***: $P < 0.001$, compared to control group.
